# Association between keratoconus and allergic conjunctivitis in children attending a Tertiary Hospital in Nigeria

**DOI:** 10.22336/rjo.2023.24

**Published:** 2023

**Authors:** Modupe Medina Balogun, Maryam Bola Fashola

**Affiliations:** * Department of Surgery, Ophthalmology Unit, Lagos State University, College of Medicine, Lagos State, Nigeria; ** Department of Ophthalmology, Lagos State University, Teaching Hospital, Lagos State, Nigeria

**Keywords:** allergic conjunctivitis, children, keratoconus, vernal keratoconjunctivitis

## Abstract

**Objective:** To ascertain an association between keratoconus and allergic conjunctivitis and to know if it is necessary to investigate all patients with allergic conjunctivitis for keratoconus.

**Methods:** A hospital-based prospective study in which the eyes of children presenting with ocular allergic diseases were examined. Social demographics and clinical data were captured in a questionnaire. All the patients underwent keratometry using the autorefractor-keratometer and the pachymeter was used to measure the central corneal thickness. Data analysis was done with IBM SPSS version 28 for Windows. Frequency and chi-square were used as descriptive statistics to determine the association between dependent and independent variables. Inferential statistics using one-way ANOVA and t-test. P - Value at <0.05 was considered statistically significant.

**Results:** 121 children with allergic conjunctivitis were reviewed. Males were 72 and females 49. The visual acuity was 6/ 6-6/ 18 in 116, and mild visual impairment - 6/ 18-6/ 60 in 5. The most common complaint was frequent itching in 109 (90.1%). Keratometry was normal in 120 (99.5%), while probable keratoconus was found by keratometry in only one patient (0.5%). Central corneal thickness was within the normal range in 33 (27.3%) children, 43 (35.5%) had thin corneas, while 45 (37.2%) had cornea thickness of more than 560 microns.

**Discussion:** Pediatric keratoconus tends to be more aggressive than adult keratoconus with an increased risk of corneal opacities and subsequent keratoplasty. As a result of these negative impacts, early detection and prompt treatment are mandatory.

**Conclusion:** The prevalence of keratoconus was not found to be high in this study population, but with facts emerging between the association of allergic conjunctivitis, and increased prevalence of keratoconus, it is pertinent to integrate keratoconus screening as part of the management of allergic conjunctivitis using an appropriate tool such as video keratography and slit lamp biomicroscope.

**Abbreviations: **KC = Keratoconus, CLEK = Collaborative Longitudinal Evaluation of Keratoconus, SAC = Seasonal Allergic Conjunctivitis, PAC = Perennial Allergic Conjunctivitis, VKC = Vernal Keratoconjunctivitis, IOP = Intraocular Pressure, CCT = Central Corneal Thickness, TNF-α = Tumor Necrosis Factor-Alpha, IL = Interleukin

## Introduction

Keratoconus (KC) is described as a condition in which the cornea assumes a conical shape with coinciding corneal stromal thinning inducing irregular astigmatism, myopia, and protrusion, leading to mild to markedly impaired visual quality [**1**]. It is a non-inflammatory, progressive thinning of the cornea, usually a bilateral, but asymmetrical disorder that is associated with changes in the structure of the collagen organization of the cornea [**2**].

Keratoconus (KC) is from the Greek word “kerato”, which means cornea, and „konos” meaning cone [**2**].

The prevalence of KC is 1:2000 in the general population, while in the pediatric population, it is 0.16% [**3**]. A study undergone in Lebanon reported a prevalence of 1:200 in children [**4**], while a more recent study in Saudi Arabia found a prevalence of 1:25 [**5**]. The variability between the results may be due to environmental factors, genetics, nutrition, and study tools employed in their study [**3**]. KC occurs in all populations, but may be more frequent in certain ethnic groups such as Indians, Pakistanis, Arabs, and Polynesians, than in Caucasians [**6**]. The occurrence of KC is greater in regions with dusty, hot, and dry climates [**7**]. Previous studies have reported greater rates of KC in males [**8**], while some found it to be greater in females [**9**] and other studies did not find any difference between males and females [**9**].

KC is most frequently diagnosed in adolescents with the onset in puberty and may progress until the third or fourth decades of life [**10**]. The youngest age of KC documented was a 4-year-old girl with Down’s syndrome [**11**].

Though less common, pediatric KC is more aggressive than adult KC [**3**,**10**,**12**] because of the dynamic environment in the young cornea, in which higher rates of corneal collagen remodeling are noticed in comparison to the adult cornea [**12**].

Not much work has been done on keratoconus in Africa, thus there is a dearth of knowledge in the epidemiology field in Africa. Despite much research, the etiology of KC is still misunderstood. It has been previously documented that a non-inflammatory process is associated with the pathogenesis of the disease [**13**], however, current studies found evidence of cytokines and inflammatory markers as interleukins (IL-1, IL-6, IL-8) and tumor necrosis factor alpha (TNF-α) in the tear-film of patients with keratoconus [**14**]. The imbalance between pro and anti-inflammatory cytokines that give rise to altered corneal structure and function is accountable for activating metalloproteinases and promoting apoptosis of keratocytes [**15**] thus, leading to collagen cross-linking causing altered corneal rigidity or biomechanical strength [**16**]. It has been proposed that the epithelial thinning in KC might occur due to the apoptosis caused by chronic epithelial injury after environmental risk factors that release apoptosis cytokines [**17**]. The causes and factors incriminated in the pathogenesis of KC include genetics, environment, enzymes, oxidative stress, and hormones, and they are all interlinked together. The environmental factors are rubbing of the eyes, atopy, and wearing of contact lenses. In the collaborative longitudinal evaluation of keratoconus (CLEK) study, hay fever or allergies were seen in 52.9% of patients with KC, 14.9% had asthma, 8.4% had atopic dermatitis, while vernal keratoconjunctivitis (VKC) was seen in 27% and 40% had abnormal topography [**3**]. A study from India that evaluated 274 patients with KC revealed a much higher prevalence of allergy in those patients [**18**]. Studies have confirmed that KC is prevalent in children with vernal keratoconjunctivitis (VKC) [**19**-**21**], though fewer studies have shown a non-conclusive association between KC and ocular allergy [**22**]. Ocular itching in the form of eye rubbing is a common occurrence in allergic conjunctivitis including vernal keratoconjunctivitis. Symptoms of dryness of the eyes and ocular irritation and fatigue can evoke eye rubbing. Mechanical rubbing of the eyes causes corneal micro trauma and thinning, which lead to a reduced corneal rigidity and corneal remodeling that can result in increased corneal curvature [**23**]. Also, eye rubbing can elevate the level of inflammatory molecules in tears including metalloproteinase (MMPs)13, tumor necrosis factor-α (TNF-α), and interleukin-6 (IL-6), and the results in stromal remodeling and keratocyte apoptosis are the main forms of organizations for cell death in keratoconus cornea [**24**]. A study undergone in 2016 in Saudi Arabia by Al-Shamman et al. showed eye rubbing to be the commonest associated factor as evident in 44.8% of KC patients seen [**25**]. Another study from Iran, undergone by Rabinowitz Y, in 2003, showed eye rubbing in 83% of keratoconus subjects compared to 58% in standard control [**26**]. In Africa, in a study undergone in Egypt by Alyaa Saeed Ahmed et al., it was estimated that keratoconus was found in 34% of the study population with allergic eye disease [**21**]. In a hospital-based study performed in Nigeria on patients with a clinical diagnosis of VKC, from 2000 to 2009, among other things, keratopathy was observed in 6 (22%) patients with one case of keratoconus [**27**]. Another mechanism that causes keratoconus in atopy patients is elevated intraocular pressure. Fluctuation in intraocular pressure (IOP) due to eye rubbing can cause the development of keratoconus by indirect traumatization of the keratocytes from the significant fluctuations in IOP [**28**,**29**]. Temperature has also been associated with corneal remodeling [**30**]. Eyelid closure can increase the corneal temperature due to the proximity of the palpebral conjunctiva and the circulatory warmth from its vasculature. An upregulation of collagenase activity related to the temperature spikes is likely to appear during the period of rubbing [**30**]. Compared to Europe and North America, the increased prevalence of KC in warm sunny regions has led to the hypothesis that too much exposure to the sun in these regions accounts for the high prevalence [**31**]. 

Keratoconus with the antecedent symptoms of blurry vision, nearsightedness, and unsatisfactory visual correction with glasses, which may warrant frequent changing, can cause poor performance in school and influence the academic and social development of the child with an undesirable effect on the well-being of the child [**12**]. Also, school non-attendance for an ocular reason was found to be 5 times more often met in children with VKC than among those without [**32**]. Pediatric keratoconus is considered to be more aggressive, with rapid progression, than in adults, as a result of the dynamic environment in the young cornea [**3**,**10**,**12**], thus, the early detection and prompt management to stop the advancement of the disease at an early stage in susceptible patients is mandatory by maintaining good spectacle-corrected visual acuity and reducing the reliance on contact lens, which is unattainable in this environment, and improve the wellbeing of the child [**10**].

This study aimed to ascertain any association between keratoconus and allergic conjunctivitis, determine whether to investigate patients with allergic conjunctivitis for keratoconus, institute treatment for KC early, and advise patients with allergic conjunctivitis to avoid eye rubbing, as eye rubbing precipitates the onset and exacerbates the advancement of KC.

## Methods

A hospital-based prospective study, in which the eyes of children presenting with ocular allergic diseases from June 2021 to June 2022 were examined. Approval for the study was obtained from the Lagos State University Teaching Hospital (LASUTH) Medical and Health Research Committee. The tenets of the Helsinki Declaration were adhered to. Social demographic and clinical data were captured in a questionnaire. All the patients had visual acuity performed with the Snellen’s chart, keratometry was done using the autorefractor-keratometer, and central corneal thickness was measured with the pachymeter. Patients were divided into three groups: vernal keratoconjunctivitis (VKC) group, seasonal allergic conjunctivitis (SAC) group, and perennial allergic conjunctivitis (PAC) group (**Table 1**). 

**Table 1:** Clinical diagnosis of children aged 5 – 15 with allergic conjunctivitis in LASUTH

		Diagnosis			
	Total	SAC	PAC	VKC	X2 (P-value)
Gender					
Male	72 (59.5)	11 (15.3)	14 (19.4)	47 (65.3)	8.246 (0.016*)
Female	49 (40.5)	17 (34.7)	12 (24.5)	20 (40.8)	
Age category					
0 -5	28 (23.1)	4 (14.3)	5 (17.9)	19 (67.9)	12.315 (0.015*)
6 -10	55 (45.5)	20 (36.4)	13 (23.6)	22 (40.0)	
11 -15	38 (31.4)	4 (10.5)	8 (21.1)	26 (68.4)	
Total	121 (100.0)	28 (23.1)	26 (21.5)	67 (55.4)	
SAC = Seasonal allergic conjunctivitis; PAC = Perennial allergic conjunctivitis; VKC = Vernal keratoconjunctivitis; * significant at p<0.05.					

Data were analyzed using IBM SPSS version 28 for Windows. Descriptive statistics were performed using frequency and chi-square to ascertain the association between dependent and independent variables. An inferential statistical analysis using one-way ANOVA and t-test with the P-value of <0.05 was considered statistically significant.

## Results

**Fig. 1:**
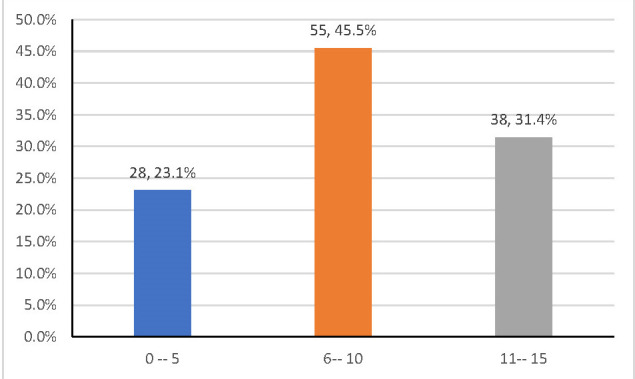
Age range of the participants

One hundred and twenty-one (121) patients with allergic conjunctivitis were reviewed. Their ages ranged from 5 to 15 years. Most of the children, 45.5%, were aged 6-10 years, while 38 (31.4%) were within the age range 11-15 years (**Fig. 1**).

**Fig. 2:**
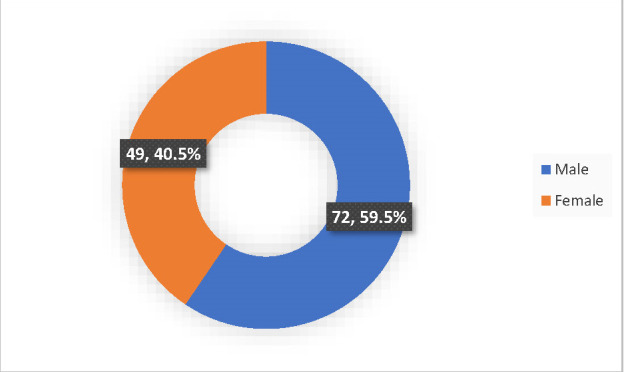
Gender distribution of the participants

Seventy-two children (59.5%) with allergic conjunctivitis were males and 40.5% were females with a ratio of 1.47:1 (**Fig. 2**).

Vernal keratoconjunctivitis (VKC) was the most common finding - 67(55.4%) (P-0.015) and more common among children between 11-15 years old (68.4%) and in males (65.3%). The visual acuity of most of the children was within the normal range, 116 (95.9%) had a visual acuity of 6/ 6-6/ 18, and 5 had a mild visual impairment between 6/ 18-6/ 60. Itching was the most common complaint in VKC - 61 (91.0%), with hyperpigmentation of the lids in 68.6% of the participants.

Conjunctiva signs were different across the different types of allergic conjunctivitis (P-0.040), 53.7% having brown conjunctiva, the majority seen in VKC, 19% being hyperemic, and 26.4% having white conjunctiva. The cornea was mainly clear in most of the children - 116 (95.9%), while 3 children had punctate epithelial keratitis and 2, shield ulcers.

Keratometry reading was normal in 120 (99.5%) of the 121 children, with only one child (0.5%) in the VKC group with a keratometry reading suggestive of probable keratoconus in the right eye (P-0.666). The child with the keratometry reading suggestive of probable keratoconus was in the 11-15 age group and a male.

Pachymetry was within the normal range in 33 (27.3%) children, the majority 15 (22.4%) in the VKC group, 10 (35.7%) in the seasonal allergic conjunctiva (SAC) group, and 8 in the perennial allergic conjunctiva group (PAC). 43 (35.5%) had thin cornea, while 45 (37.2%) had cornea thickness more than 560 microns, the most of them also in the VKC group 24 (35.8%) (P-0.226).

## Discussion

The prevalence of ocular allergic diseases is known to be increasing worldwide and is also known to cause reductions in work and educational productivity and the overall well-being of those affected [**33**]. The visual impairment caused by keratoconus may affect social and educational development in the children affected, thus negatively impacting their well-being [**12**]. Also, pediatric keratoconus has a tendency of being more aggressive than adult keratoconus, with an increased risk of corneal opacities and subsequent keratoplasty [**12**]. As a result of these, early detection and prompt treatment are mandatory. Of the 121 children examined, more male children were observed with allergic conjunctivitis, with a ratio of 1.47:1. This was comparable to findings in a study by Shi-Yao Zhang et al. [**34**], in which more males were found in the allergic conjunctivitis group. Abdus Salam Arif et al. [**35**] found a male: female ratio of 207:83, and Abiy Maru Alemayehu et al. [**36**] found a ratio of 2.2:1, though these studies looked at patients with VKC only. Contrary to some other studies [**37**,**38**] that reported more females than males, one reported an equal distribution of males and females [**39**]. The patients’ ages ranged from 0-15 years, with the majority aged between 6-10 years (45.5%). This was comparable to a research in Nigeria [**37**], in which allergic conjunctivitis was found prevalent in the younger age group. This was contrary to other studies [**38**,**40**,**41**] that reported allergic conjunctivitis in the older age group. The affectation of the younger age group could be due to their susceptibility to environmental factors that precipitate allergic conjunctivitis. More male affectation could result from more exposure to environmental pollution, as they tend to spend more time outdoors than females. Vernal keratoconjunctivitis (VKC), which is a vision-threatening form of ocular allergy, was more prevalent in this study and more in the 11-15-year age group. Patricia Maria Fernandes et al. [**42**] and Ayisha Kausar et al. [**43**] recorded a similar finding. This could be attributable to the dry, warm climate in the city, in which this study was conducted, or the older children were more tolerant to the symptoms of VKC, and thus did not seek medical attention early, although SAC is said to be generally more prevalent [**41**,**44**] and milder. Frequent itching and watering were common findings in this study, connoting the inflammation to be allergic in origin. This has been reported in many studies [**38**,**41**-**43**,**45**]. Central corneal thickness was found to be within the reference range in 27.3% (33) of the children, while 35.5% (43) had thin cornea and 37.2% (45) showed CCT of > 560 microns, more in the VKC patients (35.8%). This was contrary to a study undergone by Vijay Gautam et al. [**46**], which showed decreased central corneal thickness in the VKC subjects examined. The prevalence of keratoconus in this study was 0.5% by keratometry. This aligned with a study undergone by Wade et al. [**39**] in Gambia, which found keratoconus in 0.9% of the patients examined and the study of Roberto Caputo et al. [**47**], which found keratoconus in 0.77% of the patients. The lower prevalence might have been a result of the non-availability of sensitive instruments such as videokeratography. A higher occurrence of keratoconus was found in a study by Mugho et al. [**20**], which found that 10.6% of the population studied had keratoconus by clinical diagnosis, 14.4% by keratometry, and 30.9% by topographic criteria, and Totan et al. [**48**] found 8.5% had keratoconus by slit lamp biomicroscopy, 18.3% by keratometry and 26.2% by corneal topography.

## Conclusion

The prevalence of keratoconus was not high in this study. But, with the existing facts emerging between the association of allergic conjunctivitis, especially eye rubbing, and increased prevalence of keratoconus, it is pertinent to integrate keratoconus screening as part of the management of allergic conjunctivitis using appropriate tools like videokeratography and slit lamp biomicroscopy as pediatric keratoconus tends to be more aggressive than adult keratoconus and is also associated with rapid progression. Thus, the early detection of keratoconus in children with allergic conjunctivitis associated with eye rubbing regardless of age and any associated ocular pathology is mandatory. Also, the early diagnosis and intervention would reduce the demand for possible corneal collagen cross-linking or keratoplasty, which are not available in a country with resource deficiency like the one in which the study was carried out.

Our study had some limitations, such as the non-availability of an appropriate tool for the diagnosis of keratoconus, like videokeratography, and the follow-up of the children to 20-30 years of age, in whom the highest rate of occurrence of KC was seen.

**Conflict of Interest statement**

The authors state no conflict of interest.

**Informed Consent and Human and Animal Rights statement**

Informed consent has been obtained from all legal guardians and individuals included in this study.

**Authorization for the use of human subjects**

Ethical approval: The research related to human use complies with all the relevant national regulations, institutional policies, is in accordance with the tenets of the Helsinki Declaration, and has been approved by Lagos State University Teaching Hospital (LASUTH) Medical and Health Research Committee, Lagos State, Nigeria.

**Acknowledgements**

None.

**Sources of Funding**

None.

**Disclosures**

None.
